# Multimodality Imaging Approach to Spontaneous Coronary Artery Dissection

**DOI:** 10.3390/jcm12010154

**Published:** 2022-12-25

**Authors:** Gemma Marrazzo, Stefano Palermi, Fabio Pastore, Massimo Ragni, Mariarosaria De Luca, Michele Gambardella, Gaetano Quaranta, Giancarlo Messalli, Lucia Riegler, Valeria Pergola, Andrea Manto, Antonello D’Andrea

**Affiliations:** 1 Department of Cardiology, Umberto I° Hospital, 84014 Salerno, Italy; 2 Public Health Department, University of Naples Federico II, 80131 Naples, Italy; 3 Department of Translational Medical Sciences, University of Naples Federico II, 80131 Naples, Italy; 4 Department of Advanced Biomedical Sciences, University of Naples Federico II, 80131 Naples, Italy; 5 Department of Radiology, “Villa Malta” Hospital, 84087 Salerno, Italy; 6 Department of Cardiology, Padua University Hospital, 35128 Padua, Italy; 7 Department of Neuroradiology, Umberto I° Hospital, 84014 Salerno, Italy

**Keywords:** spontaneous coronary artery dissection, multimodality imaging, coronary imaging, myocardial infarction

## Abstract

Spontaneous Coronary Artery Dissection (SCAD) refers to the spontaneous separation of the layers of the vessel wall caused by intramural hemorrhage, with or without an intimal tear. The “typical” SCAD patient is a middle-aged woman with few traditional cardiovascular risk factors, and it’s frequently associated with pregnancy. Because of its low incidence, its pathophysiology is not fully understood. SCAD presents as an acute coronary syndrome, with chest pain, dyspnea, syncope, or heartbeat, even if diagnosis and clinical handling are different: coronary angiography is currently the main tool to diagnose SCAD; however, in doubtful cases, the use of both invasive and noninvasive cardiovascular imaging methods such as intravascular ultrasound or optical coherence tomography may be necessary. This paper aims to review the current state of knowledge on SCAD to address its demographic features, clinical characteristics, management, and outcomes, focusing on diagnostic algorithms and main multimodality imaging techniques.

## 1. Introduction

Spontaneous Coronary Artery Dissection (SCAD) refers to the spontaneous separation of the layers of the vessel wall caused by intramural hemorrhage, with or without an intimal tear. This condition is not associated with trauma, atherosclerosis or iatrogenic causes and may be an expression of an underlying systemic arterial disease, namely, fibromuscular dysplasia [1,2,3].

SCAD has been recognized as one of the main reasons for acute coronary syndrome (ACS). It predominantly affects young or middle-aged women including a small proportion who are pregnant or post-partum. Although SCAD is uncommon, accurate diagnosis is critical as optimal management differs from atherosclerotic ACS. Angiographic appearances of SCAD are usually definite; however, ambiguous cases may require further investigation [1,2,3].

The present paper aimed to review the current state of knowledge on SCAD to address its demographic features, clinical characteristics, management, and outcomes, focusing on diagnostic algorithms and main multimodality imaging techniques.

## 2. Epidemiology

### 2.1. Sex, Age, Ethnicity

The real incidence of SCAD is difficult to define as it is often underestimated. Although SCAD is an uncommon cause of all heart attacks (<1% of all acute myocardial infarctions are caused by spontaneous dissections) [3], its incidence in acute myocardial infarctions in women remains considerable. It is estimated that about 1/3 of acute myocardial infarctions in women <50 years are caused by spontaneous dissections [4,5]. The “typical” SCAD patient is a middle-aged woman with few traditional cardiovascular risk factors, albeit ACS due to SCAD has been observed from the late teens to the ninth decade of life [5]. SCAD is likely influenced by a combination of factors that include sex; hormonal fluctuations; underlying arteriopathies; genetics; and environmental, physical, and emotional precipitants. Women comprise 87% to 95% of SCAD with a mean age of presentation between 44 and 53 years [6,7,8]. Data on SCAD in men are limited because the prevalence of this condition among men is low. SCAD in men is more frequently associated with excessive physical exertion (e.g., exercise or heavy lifting) and tends to have a lower prevalence of fibromuscular dysplasia than in women with SCAD [3].

White Caucasian ethnicity predominates in most observational series; however, cases have been described in many racial groups [5]. SCAD fatalities are uncommon; however, their incidence remains unknown, due to challenges with accurate post-mortem diagnosis [1].

### 2.2. Pregnancy-Associated SCAD

The strong female sex predominance of SCAD and the association with pregnancy and perhaps multiparity strongly suggest some association between female sex hormones and the pathophysiology of SCAD. However, the precise relationship remains unknown, and SCAD has been described in men, post-menopausal and nulliparous women. SCAD has been described in patients taking hormonal contraception and hormone replacement therapy. However, it has never been demonstrated that exogenous hormone use is more prevalent in SCAD or that continuing to take hormones after SCAD increases the risk of recurrence [5]. Pregnancy-associated SCAD (P-SCAD; usually defined as SCAD occurring during gestation or within 12 months of delivery) accounts for approximately 5–10% of cases of SCAD. Reportedly accounts for 10–22% of ACS events in pregnancy and 23–67% of postpartum ACS [2,4,5]. There is growing evidence that P-SCAD is associated with a more severe phenotype with proximal and extensive dissections and larger infarcts. SCAD has also been observed in association with multi-parity and pre-eclampsia in some studies [9,10,11,12].

### 2.3. Inflammatory Conditions

Even if SCAD is not frequently related to systemic inflammatory disorders, laboratory testing to rule out autoimmune diseases may be taken into consideration in the post-acute setting of patients symptomatic of rheumatologic conditions [4].

### 2.4. Inheritance and Genetics

SCAD occurs rarely in patients with known hereditary conditions.

Familial SCAD is rare [13], and pathogenic variants were identified in only 3.5% of unselected patients in a genome sequencing study, suggesting that most cases are sporadic; therefore, at present, testing genetic is not recommended in all cases of SCAD [14].

### 2.5. Risk Factors for Ischemic Heart Disease

Although the prevalence of classic risk factors for ischemic disease is low, coronary dissection is more frequently associated with arterial hypertension and less frequently with diabetes mellitus [15,16,17]. Indeed, whilst risk factors are less common in SCAD patients than in atherosclerotic patients, SCAD should not be excluded from diagnostic consideration when risk factors are present.

The left anterior descending coronary artery is involved in about 40% of SCAD [18,19], mainly in its mid-to-distal segments. Other vessels or multivessel SCADs are less common [4,20].

## 3. Pathophysiology

SCAD is an acute coronary event linked to the formation and expansion of an intramural hematoma that determines the separation of the intima or intima-media complex from the underlying vessel lumen creating a false lumen that compresses the true light. This process determines, depending on the degree of compression of the true lumen, ischemia, or myocardial infarction. Two mechanisms have been hypothesized to explain the entry of blood into the vessel wall [21,22]:

Inside-out mechanism: Rupture of the endothelium with the passage of blood from the true lumen to the subintimal space.

Outside-in mechanism: Formation of “de novo” hematoma at the level of the middle tunic due to rupture of the vasa vasorum or by dissection of the medium tunic with a consequent false lumen. Several pieces of evidence, acquired with the IVUS/ OCT method, support this hypothesis. 

Frequently, there is no communication between the true and false lumen and when fenestrations are created, these derive from the breaking of the false lumen into the true lumen and not vice versa; in addition, serial angiography performed during SCAD have shown that the intramural hematoma precedes the intimal dissection [21,22,23,24]. More rarely, the occlusion of the true lumen may be due to thrombosis generated by the exposure of the pro-thrombogenic subendothelium following injury to the intima.

To explain the pathogenesis, predisposing conditions have been identified that cause the weakening of the vascular wall and precipitating conditions that, by increasing the wall stress, favor its rupture. There is a complex relationship between a vulnerable subject and potential triggers that initiate a spontaneous arterial tear or intramural hematoma (Table 1)

Among the predisposing conditions, the most frequent is fibromuscular dysplasia (FMD), a non-atherosclerotic segmental idiopathic disease of the medial tunic of the arterial walls manifested by stenosis, aneurysms, tortuosity, or spontaneous dissections of small and medium arteries caliber. FMD is linked to the proliferation of smooth muscle cells and fibrous tissue [25]. The association of SCAD with multifocal FMD in arteries has been the most frequently found and coronary dissection could be the first manifestation of FMD [20]. Multifocal FMD is defined from an angiographic point of view as areas of alternating stenosis and dilatation. There is a high prevalence of concomitant extra coronary arterial anomalies in registries of SCAD patients. In patients without diagnostic features of FMD, other arterial abnormalities have been reported, such as dissections, aneurysms, or arterial tortuosity (78%) [26,27,28].

About 80–90% of patients with FMD are female (prevalence about 4% of the population). The presence of any extracoronary vascular abnormalities (EVA) in patients with SCAD is high and includes aneurysms and noncoronary dissections. The cerebral aneurysm has been detected in 7 to 14% of patients with SCAD who have undergone screening [29,30,31].

Whether or not SCAD is a coronary manifestation of fibromuscular dysplasia or a unique but related entity with a considerable number of arterial features in common, SCAD may be a *forme fruste* of an underlying systemic arteriopathy that leaves the affected patient vulnerable to dissection when exposed to arterial shear stress related to an inciting trigger [3].

Regardless of the vascular district involved at the onset of the disease, all patients with FMD should evaluate at least in one arterial district “from brain to pelvis” with angioCT or MRI to allow a correct diagnosis and adequate follow-up. The same type of screening is recommended for patients diagnosed with SCAD, as the coronary disease is associated with lesions in other extra coronary areas in 45–60% of cases [25].

SCAD is frequently described in association with increased emotional stress, particularly in women [32,33,34]. This is difficult to control as emotional stress is highly prevalent in the population. However, a recent Dutch study restricted to female patients confirmed significantly higher odds of an emotional precipitant in the 24 h before SCAD (56%) than before atherosclerotic ACS (39%) [35].

An association with exercise reportedly occurs more commonly in men [35], in some cases, symptom onset coincides with isometric or unusually intense exercise, and occur in people who have a higher performance status but without a clear temporal link between an exercise episode and symptom onset.

## 4. Clinical Presentation

The clinical presentation of SCAD is similar to that of atherosclerotic ACS, with the primary difference being the patient phenotype (Table 2). Serially measured high-sensitivity biomarkers (Troponin) are therefore normally elevated, except in very rare cases with a staggered onset presentation where the timing of first symptoms and Troponin testing are not synchronous [31].

Clinical manifestations are related to three elements:

1. Extent of dissection;
2. Extent of the reduction of the vessel lumen;
3. Site.

The main symptom is chest pain which can be associated with dyspnea, syncope, or heartbeat [32]. The clinical manifestation in almost all SCAD are acute coronary syndromes; of these about ¼ are STEMI and ¾ NSTEMI; in 5% of cases, the onset may be cardiac arrest, and in 2% of cases it may be a cardiogenic shock [3].

## 5. Diagnosis

Patients with SCAD are at risk of receiving alternative diagnoses and of being discharged after emergency department evaluation because their relatively young age and absence of atherosclerotic risk factors frequently do not fit the expected phenotype of an atherosclerotic patient with MI. Accurate diagnosis of SCAD in the early stages of ACS presentation is important because management and investigation are different from those for atherosclerotic forms of coronary artery disease. Both invasive and noninvasive cardiovascular imaging techniques are integral to the diagnosis, characterization, and management of patients with SCAD. Moreover, multimodality imaging is critical for ongoing efforts to elucidate the cause and mechanism of SCAD, as often happens with other cardiovascular diseases [33].

SCAD should be suspected in all young women presenting with acute myocardial infarction in the absence of classic cardiovascular risk factors.

Differential diagnosis:

- SCA on an atherosclerotic basis;
- Coronary spasm;
- Takotsubo syndrome;
- Coronary thromboembolism;
- MINOCA.

### 5.1. Angiography

**Figure 1:**
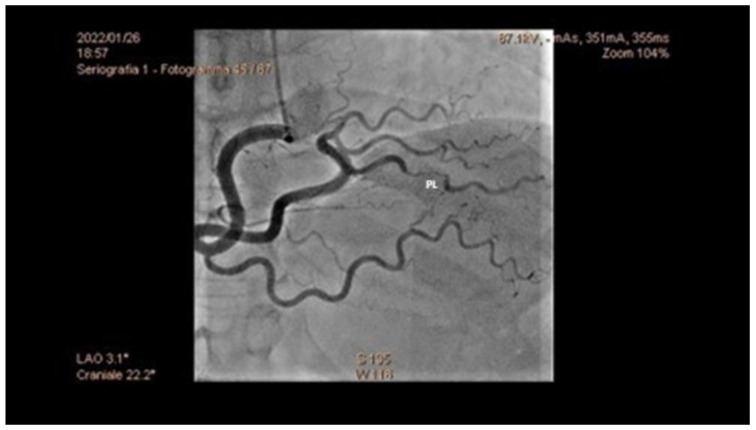
Coronary angiography of a 43-year-old woman, showing SCAD of the postero-lateral artery (PL). SCAD: spontaneous coronary artery dissection.

**Figure 2:**
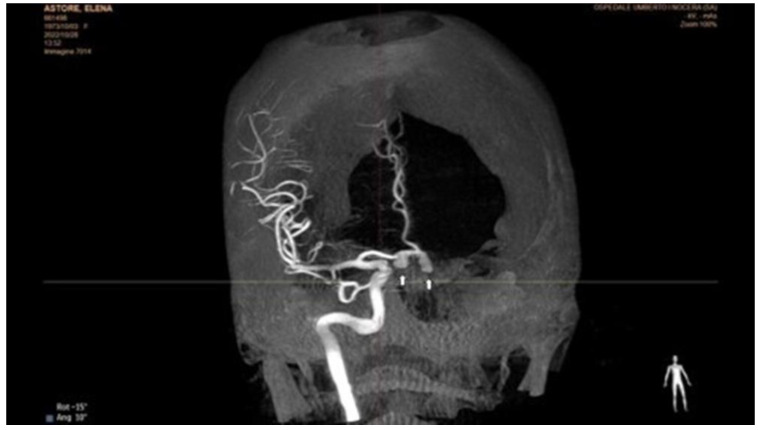
Selective rotational angiography of the right internal carotid artery. The exam shows two aneurysmal dilatations, one of which is sac-shaped in the portion of the right midsection, and the second in correspondence with the ipsilateral A1–A2 angle. Both have the bottom of the aneurysm directed downwards. The first extension has a large collar, and the second of 1–2 mm.

Coronary angiography is currently the main diagnostic tool for diagnosing SCAD. If there is a suspicion of coronary dissection, angiography should be performed as soon as possible (with timing by the guidelines for ACS based on the clinical presentation); timely diagnosis is crucial in these patients as there are important differences in the management of infarction caused by spontaneous dissection compared with that on an atherosclerotic basis. This technique is still the first-line diagnostic imaging method due to its widespread availability and recommendation for an early invasive evaluation of ACS [4] (Figure 1 and Figure 2).

One of the limitations of angiography is that it is 2-dimensional “lumenography” and does not specifically investigate the arterial wall. Angiographic aspects suggestive of dissection are the persistence of the contrast medium at the level of the arterial wall, the presence of areas of the radiolucent filling defect (“pathognomonic” but is present in only a small proportion of SCAD cases), the homogeneous reduction of the vessel lumen, the high tortuosity of the coronary vessels, the preferential involvement of the distal segments of the vessels (as opposed to atherosclerotic lesions), the absence of coexisting atherosclerosis in coronary arteries not affected by the acute process.

Coronary angiography is considered the reference method for the diagnosis of SCAD as it allows the diagnosis to be made by evaluating the angiographic data in a specific epidemiological context. However, it should be emphasized that this invasive procedure may be associated with a higher risk of iatrogenic dissection in patients with SCAD than in atherosclerotic patients as it is performed on patients with already intrinsic vessel fragility (about 2–3% risk of iatrogenic dissection is reported vs. 0.2% risk in atherosclerotic patients; this risk rises to 14% during interventional procedures) [36].

A SCAD angiographic classification is frequently used in clinical practice [37]:

TYPE 1: An intimal lesion with a contrast passage between the true and false lumen is identified. Type 1 spontaneous coronary-artery dissection (SCAD) has the pathognomonic angiographic appearance of arterial dissection, including multiple radiolucent lumens due to an intimal tear that causes contrast dye to penetrate through two flow channels. A radiolucent flap separating the two flow channels is visible on the angiography (left image, arrow). Type 1 SCAD also can result in the retention of contrast dye within the intimal tear or slow clearing of contrast material. On optical coherence tomography (OCT), an intimal tear (right image, arrow) separating the true lumen from the false lumen (FL) is shown. Asterisks indicate a guidewire shadow artifact, whereas double-headed arrows indicate an intramural hematoma.

TYPE 2: It is the most common form (from 60 to 75%). It is characterized by the absence of an intimal scar and appears as a long, diffusely stenotic segment; it is caused by the compression of the true light by the false light. They are long lesions (>20 mm) and often appear as a sudden reduction in the caliber of the vessel which can affect the central part (between two parts of normal caliber: type IIa) or extend to the end of the vessel (type IIb). Type 2 SCAD is characterized by the absence of an intimal tear and appears as a long segment of a diffusely narrowed artery because of an intramural hematoma that causes stenosis of varying severity. Type 2 SCAD lesions are long (typically >20 mm), often appear as an abrupt caliber change in the artery (angiographic images, square brackets), and will either be flanked by an artery of normal caliber (type 2A) or continue to the tip of the artery (type 2B). OCT imaging shows an intramural hematoma.

TYPE 3: Stenotic segment < 20 mm, caused by the compression of the true lumen by the false lumen. Enter differential diagnosis with atherosclerotic plaques. It should be suspected when there is a high probability of SCAD due to the patient’s phenotype, due to the absence of atherosclerotic lesions elsewhere, or in the presence of linear stenosis within tortuous segments. The type 3 variant is the least common type of SCAD and the most challenging type to recognize in angiography. The appearance also suggests compression by an intramural hematoma; however, type 3 SCAD is usually 20 mm or less in length. Type 3 SCAD is described as an atherosclerosis mimic, and intracoronary imaging is often necessary to confirm the diagnosis. This type should be considered when there is a high index of suspicion for SCAD, atherosclerosis is absent in the remainder of the coronary vasculature, the lesion is linear and long (11 to 20 mm), or coronary tortuosity is present. As with type 2 SCAD lesions, OCT imaging reveals a compressive intramural hematoma.

TYPE 4: Complete occlusion of the vessel. Differential diagnosis with thromboembolic occlusion. Sometimes the occlusion is preceded by a thinning of the vessel which may suggest the presence of an intramural hematoma. Finally, type 4 SCAD has been described as a complete occlusion of the vessel (arrow).

Simultaneous multivessel dissections are defined as dissections in more than one coronary; this occurs in around 10% of cases at presentation [2,4]. Multivessel forms are, therefore, not so rare that extreme caution is recommended during angiography evaluating each vessel in patients diagnosed with SCAD. There are no current specific studies of multi-vessel SCAD; however, the approach to management for multi-vessel SCAD should not differ from single vessel disease with each lesion being assessed for intervention on its merits and a preference for conservative management where possible [2].

Additional possible angiographic findings [2]:

- Increased coronary tortuosity;
- Predilection for more distal coronary segments;
- Predominant involvement of the left anterior descending coronary artery;
- False lumen starting and/or ending at a side branch;
- Absence or reduced incidence of co-existent atherosclerosis;
- Association with FMD;
- Association of sites of dissection with myocardial bridging.

### 5.2. Challenging Diagnoses: Role of Intravascular and Non-Invasive Imaging

Especially in doubtful cases (SCAD with the angiographic appearance of type 2–3), for diagnostic confirmation, it may be necessary to resort to the use of invasive methods such as intravascular ultrasound (IVUS) or Optical Coherence Tomography (OCT). IVUS and OCT can clarify the ambiguous appearance of SCAD [38] and both techniques can visualize the coronary intima, media, and adventitia with the identification of intramural hematoma and intimal disruption.

**Figure 3:**
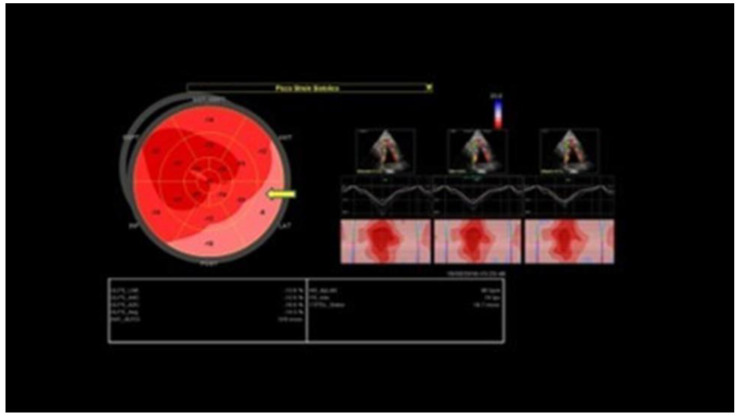
Bull’s eye representing a two-dimensional longitudinal strain of the same patient in Figure 1, with impairment of myocardial deformation in postero-lateral walls.

Before cardiac catheterization, an echocardiogram may influence pre-test probability, showing regional wall motion abnormalities or reduced strain (Figure 3) in the involved coronary territory, but its role in differential diagnosis and multimodal imaging is very limited [39].

Tweet et al. analyzed 92 echocardiograms performed for the assessment of acute SCAD and highlighted regional wall motion abnormalities (67% of patients) and preserved left ventricular ejection fraction. Despite a high prevalence of fibromuscular dysplasia in the study population, the dilation of the mid-ascending aorta and the presence of a bicuspid aortic valve were uncommon [40].

Intravascular ultrasound (IVUS) is an imaging method that uses a particular guide with an ultrasound probe connected to the distal end of the guide. It provides grayscale images of the coronary vessel and wall via a catheter with an ultrasound tip [41]. IVUS is a familiar technique that is widely available, provides a satisfactory depth of visualization, and does not require contrast agents. It allows information on the extent of the hematoma and the false lumen but the intima layer and the separation between the false and true lumen are not accurately visualized. However, it has limited resolution, which can result in diagnostic uncertainty; moreover, the false lumen’s grayscale appearances with IVUS are not sufficient to distinguish SCAD from lipid-rich atherosclerotic plaque, which is frequently a critical differential diagnosis.

Optical Coherence Tomography (OCT) is an endovascular imaging modality that provides high-resolution, high-sampling rate images of coronary tomography sections. It uses infrared light carried by an optical fiber inside a catheter. The light that illuminates the vessel lumen rotates rapidly and the simultaneous injection of contrast medium allows the light to interact with the surrounding vascular structures without interference. Vessel tissues and structures absorb and reflect light differently according to their composition. The intensity and attenuation of the recaptured optical signal are the basis of the tissue characterization carried out by the OCT. It allows the visualization of the intima tunic with any lesions; the false lumen and intramural hematoma and it is considered the most specific invasive imaging method for SCAD diagnosis confirmation. OCT also can give insight into the structure of the vasa vasorum and further elucidate SCAD mechanisms [42].

Despite the importance of intracoronary imaging, these methods should be reserved for doubtful cases and preferably for the proximal tracts of the vessels (considering that the diameter of the vessel must be large enough to allow the safe passage of the probe). Indeed, these methods are not free from risks such as the extension of the coronary dissection with the passage of the guide into the false lumen, an iatrogenic dissection from a guide catheter with consequent complete occlusion of the vessel, an extension of the dissection after injection of the contrast medium; therefore, their use must be carefully considered.

In uncertain cases of SCAD, such as those patients with a borderline troponin elevation and nonspecific findings on electrocardiogram, other imaging modalities such as coronary computed tomographic angiography (CCTA), echocardiography, myocardial perfusion imaging (MPI), or cardiac magnetic resonance (CMR) may suggest myocardial ischemia or infarction, which may then lead to CA and accurate diagnosis. Examples of this include regional wall motion abnormalities on echocardiography, decreased myocardial perfusion on coronary CTA, left ventricle dysfunction, and identification of myocardial infarction by late gadolinium enhancement on CMR [38].

**Figure 4:**
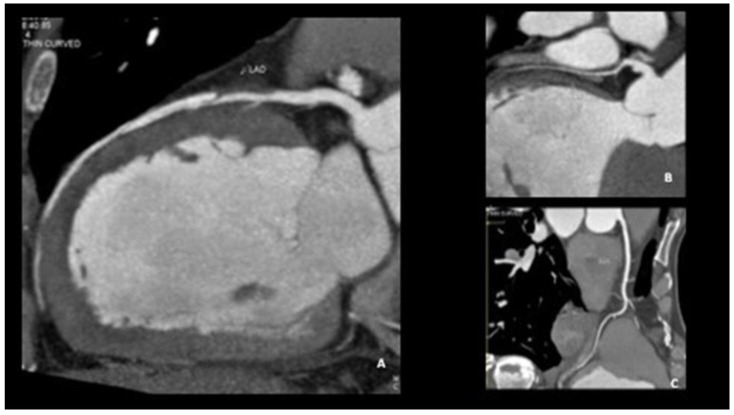
Coronary CT of a 42-year-old woman with STEMI, showing SCAD of the middle tract of LAD (**A**) and normal morphology of left circumflex (**B**) and right coronary artery (**C**). CT: computer tomography; STEMI: ST-elevation myocardial infarction; SCAD: spontaneous coronary artery dissection; LAD: left anterior descending.

**Figure 5:**
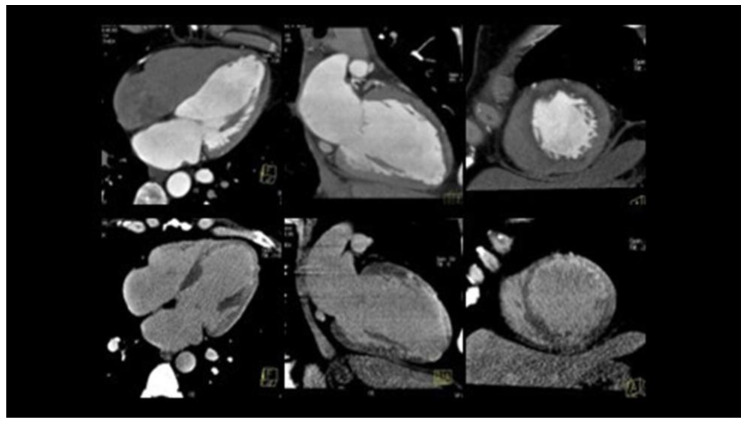
Cardiac CT of the same patient in Figure 4, showing contrast enhancement of the LV anterior wall, secondary to myocardial infarction. CT: computer tomography; LV: left ventricle.

Coronary computed tomographic angiography (CCTA) is a noninvasive diagnostic imaging technique mainly used to characterize coronary and cardiac anatomy. In atherosclerotic disease, coronary CTA demonstrates sensitivity and specificity as high as 94% and 83%, respectively, for the identification of atherosclerotic stenoses greater than 70% when compared to CA [43]. It has been applied both in the initial diagnosis of SCAD and in the follow-up; however, cardiac CTA diagnostic criteria for SCAD need further refinement. CCTA can be used to visualize dissection, flaps, stenoses, and intramural hematomas (which have been described as a “sleevelike” wall thickening); CCTA allows the visualization of the leakage of contrast medium in the false lumen and compared to coronary angiography excludes the likelihood of iatrogenic vessel damage. CCTA is thus an attractive form of noninvasive ancillary imaging when the diagnosis of SCAD is uncertain on catheter-based angiography, particularly in cases of proximal lesions. However, there are limitations to CCTA in the diagnosis of SCAD. During the acute SCAD episode, an intramural hematoma can sometimes be visualized on the CCTA in SCAD patients, but this can be challenging since the hematoma can mimic the appearance of artifact due to cardiac motion in the central coronary arteries or of adjacent myocardium in distal branch vessels. Moreover, noncalcified atherosclerotic plaque can be mistaken for an intramural hematoma, and the spatial resolution of CCTA for small vessels limits visualization of the distal portion of the vessels that are often affected by SCAD. As a result of these technical and interpretation limitations, coronary arteries may be reported as normal on coronary CTA in SCAD patients, and a negative coronary CTA does not fully exclude SCAD. When cardiac biomarkers are elevated or SCAD is suspected in the acute setting, patients should undergo CA to identify the culprit. After the initial diagnosis of SCAD, coronary CTA may be a worthwhile alternative to invasive CA to assess vessel healing, particularly if a patient continues to report chest pain (Figure 4 and Figure 5).

CMR is a noninvasive, non-radiating imaging modality that can assess cardiac anatomy, ventricular function, myocardial perfusion, and characterization by late gadolinium enhancement for the detection of ischemia/infarction, inflammation, or fibrosis [44]. Imaging showing delayed gadolinium enhancement in a territory corresponding to a suspected dissection can help confirm SCAD or suggest an alternate diagnosis (e.g., myocarditis), but a normal CMR does not exclude SCAD. In a substantial minority of patients with angiographically confirmed SCAD, there is no evidence of myocardial infarction on CMR [5]. Furthermore, coronary magnetic resonance angiography could be a potential tool for noninvasive visualization of the coronary artery lumen without the use of ionizing radiation. However, the technique is yet limited by long image acquisition time, low spatial resolution, and operator dependency.

### 5.3. Gray Diagnostic Zones

Some angiographic pictures can “mimic” the initial diagnosis of SCAD [1].

1. Streaming of contrast: it can give an image similar to a linear type I dissection, but it disappears as the injection pressure of the contrast medium is increased.
2. Coronary vasospasm: it disappears with an injection of nitroglycerin ic.
3. Iatrogenic dissections: in this case, the differential diagnosis between SCAD and iatrogenic dissection is given by the ECG and symptoms preceding the chorus; in the case of iatrogenic dissection, symptoms and ECG changes appear during coronary angiography.
4. Coronary embolism: Type 4 SCAD and coronary embolic occlusion can look very similar. The truncation of multiple branches due to a shower of embolic material can be a diagnostic feature but needs to be carefully distinguished from multivessel SCAD. Non-occlusive embolic clots can sometimes cause a filling defect, giving an apparent dual lumen appearance which mimics Type 1 SCAD.
5. Rupture or erosion of atherosclerotic plaque with the penetration of contrast medium inside the plaque. These may include non-obstructive sites of plaque erosion and rupture where contrast penetration of ruptured plaque or a tongue of thrombus can give a linear appearance; sometimes embolization of thrombus into the distal vessel can give a dual lumen appearance distally.
6. Takotsubo syndrome: the apical left anterior descending coronary artery is a common site for SCAD. This can lead to an apical regional wall motion abnormality similar in appearance to Takotsubo syndrome. Both conditions can also lead to the complete recovery of heart function.

In cases of diagnostic uncertainty:

- Reconsider the pre-test probability.
- Check for true lumen thrombus. SCAD does not appear to be highly thrombogenic and the presence of substantial true lumen thrombus should raise suspicion of an alternative diagnosis.
- Consider intracoronary (OCT) imaging.
- Assess pre-demission or evidence of atherosclerosis with follow-up CT Coronary Angiography.
- Consider other tests to assess the probability of paradoxical embolism or intracardiac thrombus [1].

As frequently happens in cardiology, multi-imaging is often the winning choice (Table 3).

### 5.4. Diagnostic Algorithm

**Figure 6:**
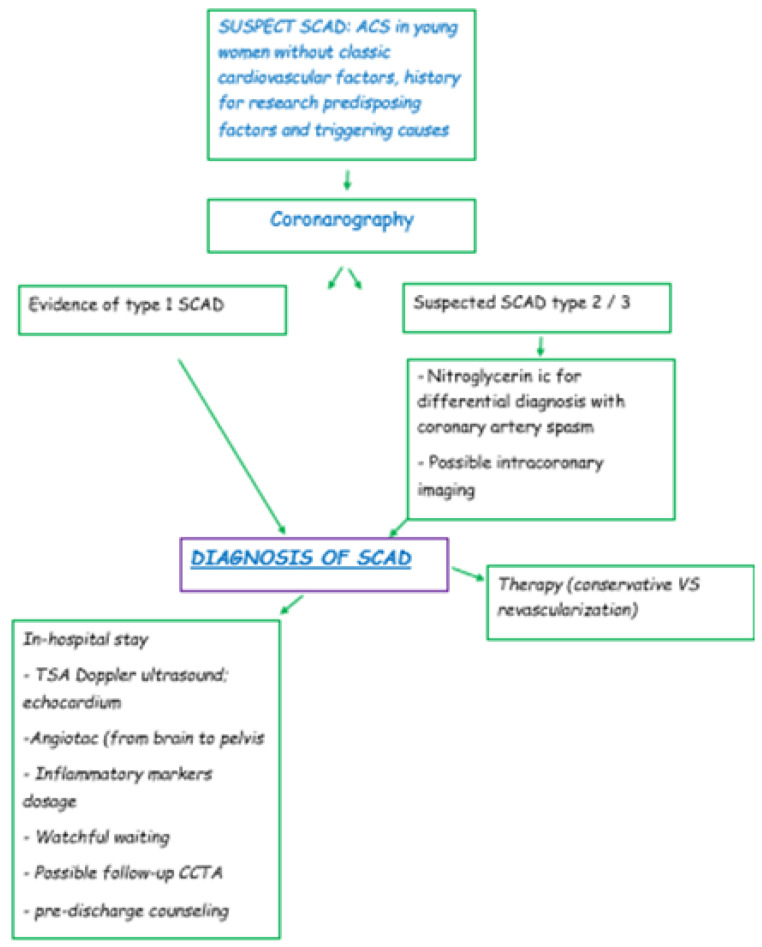
Diagnostic algorithm of a suspected SCAD. SCAD: spontaneous coronary artery dissection; ACS: acute coronary syndrome; IC: intracoronary; TSA: supra-aortic trunk; CCTA: cardiac coronary tomography angiography.

Figure 6 shows the diagnostic pathway of a suspected SCAD.

## 6. Management

### 6.1. Acute Management

There are currently no randomized comparative studies available on the best treatment for SCAD. The acute phase management goal is to restore or preserve myocardial perfusion and cardiac function.

The therapeutic strategy is conditioned by:

1. Clinical presentation of onset;
2. Hemodynamic picture of the patient;
3. Angiographic characteristics of the lesion;
4. Extension of the myocardial territory at risk.

Angiographic follow-up series show most SCAD managed conservatively will heal with the restoration of normal coronary architecture. Moreover, several studies suggest most SCAD cases managed conservatively heal completely within 3–6 months of the index event [45]. For these reasons, expert consensus (not randomized trial data) suggests that medical management is preferred over immediate revascularization for patients who are in clinically stable condition [2,4].

The use of the revascularization procedure is reserved for patients with high-risk clinical or angiographic characteristics. Clinical features that identify high-risk patients are the persistence or recurrence of chest pain, hemodynamic instability, cardiogenic shock, and ventricular arrhythmias [3]. The angiographic features that identify high-risk patients are multivessel coronary dissections, those involving CT or the ostium of the IVA, occluded vessels, and STEMI presentations [11].

The TIMI flow downstream of the dissection and its extension are important elements that are taken into consideration. Where PCI is undertaken, the goals of revascularization may differ from PCI in the context of atherosclerosis, to restore flow rather than restore a normal coronary architecture; in this case, the target is to maintain a TIMI II/III flow even in the presence of significant stenosis [9,38,45]. Interventional PCI strategies include long drug-eluting stents, direct stenting without balloon predilation, balloon angioplasty, cutting balloon fenestration, multi-stent approach, and bioresorbable stent. Despite the risk of complications, in SCAD-PCI cases with reduced TIMI flow at presentation, improvements in coronary flow are reportedly achieved in 84% (with worsening in only 7%), and medium-term outcomes in terms of MACE and left ventricular function are good [46].

Overall, recurrent cardiovascular events are frequent after an initial SCAD, with rates of repeat coronary dissection ranging from about 20% in long-term follow-up studies.

### 6.2. Short-Term Management

Ongoing prospective trials should further elucidate the medical management of this challenging condition. Usually, after PCI, the recommended therapy is dual antiplatelet, rather than systemic anticoagulation; statins are not routinely used.

Due to the non-clear benefits of clopidogrel in SCAD, its use during in pregnant women not undergoing PCI must be carefully evaluated [47], since low doses of aspirin be safer in this population. Even if β-blockers are linked to fetal growth restriction, labetalol is frequently used for pregnant women with hypertension, while others should be avoided even during breastfeeding due to possible placental alterations [48].

## 7. Cardiac Rehabilitation and Physical Activity after SCAD

Cardiac rehabilitation after SCAD-related MI is strongly recommended due to its important role in secondary prevention and psychosocial support. This should have a multidisciplinary approach including exercise rehabilitation, psychosocial counseling, dietary and cardiovascular disease education, and peer group support [49,50]. An individualized, wise, and physician-prescribed approach to physical activity is essential to these patients to reduce symptoms, provide physical and mental benefits, and improve their quality of life. Avoiding high-intensity activity, breathing correctly (avoid Valsalva maneuver) and resting between series, choosing comfortable settings, warming up and cooling down and following trainer advice are all fundamental aspects of their training [4,51].

## 8. Conclusions

Timely SCAD diagnosis is crucial as there are important differences in the management of infarction caused by dissection compared with atherosclerotic ischemia. Invasive coronary angiography allows for diagnosis in most patients with SCAD. However, in some doubtful cases, it may be necessary to use other invasive and noninvasive cardiovascular imaging to confirm the diagnosis of SCAD. Further studies should be performed to better define risk stratification scores in SCAD, to validate diagnostic algorithms and use of emerging imaging modalities. Optimal medical management and follow-up should be tailored to the individual’s profile.

## Figures and Tables

**Table 1:** Predisposing and precipitating conditions of spontaneous coronary artery dissection adapted from [18].

Predisposing Conditions	Precipitating Conditions
Disorders of the connective tissue: - Fibromuscular dysplasia - Marfan syndrome - Ehlers–Danlos syndrome - Cystic necrosis of the media - Systemic inflammation - Systemic lupus erythematosus - Rheumatoid arthritis - Churg-Strauss syndrome - Chron’s disease - Polyarteritis nodosa - Sarcoidosis - Pregnancy - Prepartum (within 6 weeks of delivery) - Postpartum with late occurrence (from 6 weeks to 12 months from childbirth) - Postpartum with very late occurrence (12 to 24 months from childbirth) Coronary spasm	Intense physical exercise (isometric or aerobic activity) Intense emotional stress Childbirth and labor of childbirth Intense Valsalva-like activity (retching, vomiting, coughing, bowel movements) Drugs (cocaine, amphetamines, methamphetamines) Hormonal therapies (injection of β-chorionic gonadotropins, corticosteroid injections)

SCAD: Spontaneous coronary artery dissection.

**Table 2:** Clinical symptoms of spontaneous coronary artery dissection (adapted from 18).

Clinical Presentation	Incidence (%)
Chest pain	91–100%
Dyspnea	25–57%
Syncope	23%
Malignant ventricular arrhythmias	14%
Bradyarrhythmia	Rare
Cardiogenic shock	Rare
Unexpected death	Rare
Asymptomatic with random findings on coronary angiography	Rare

**Table 3:** Summary of multi-imaging techniques in SCAD adapted from [38].

	Coronarography	IVUS	OCT	CCTA
Advantages	Easily executable Images suggestive of SCAD in most cases	Visualization of the intramural hematoma and the flap	Visualization of the vessel wall, the intimate, the false light, intramural hematoma, and any flaps and areas of communication between the true and false lumen	No possibility of iatrogenic damage Image of the presence of contrast in the false lumen Visualization of intramural hematoma
Limitations	Invasive Image of the vase light but not the wall	Passage of the guide in the coronary artery No identification of the middle intimal membrane No identification of small areas of connection between false and true lumen	Passage of catheter in coronary artery and injection of contrast at high pressure, with the danger of expansion of the dissection itself	Limited data on its use in acute Absence of specific diagnostic criteria for EXP Frequency-dependent motion artifacts Limited utility for pots < 2.5 mm in diameter Difficulty in distinguishing between non calcified atherosclerotic plaques and intramural hematoma
Use	Diagnosis of SCAD	Confirmation of diagnosis	The gold standard method for the confirmation of SCAD	Follow up

IVUS: intravascular ultrasound; OCT: optical coherence tomography; CCTA: coronary computer tomography angiography; SCAD: spontaneous coronary artery dissection.

## Data Availability

Not applicable.
